# Optimisation of enzymatic hydrolysis of cassava peel to produce fermentable sugars

**DOI:** 10.1186/s13568-015-0146-z

**Published:** 2015-09-17

**Authors:** Richard Bayitse, Xiaoru Hou, Anne-Belinda Bjerre, Firibu Kwasi Saalia

**Affiliations:** Council for Scientific and Industrial Research/Institute of Industrial Research, P.O Box LG 576, Legon, Ghana; Danish Technological Institute, Gregersensvej 1, 2630 Taastrup, Denmark; Nutrition and Food Science Department, University of Ghana, P.O Box LG 25, Legon, Ghana

**Keywords:** Enzymatic hydrolysis, Glucose, Cassava peel

## Abstract

Enzymatic hydrolysis of cassava peels was evaluated using cellulase and beta-glucanase enzymes and their mixtures at three different enzyme loadings with time. The pH of the medium used for hydrolysis was 5 and the temperature was 50 °C. The efficiency of the hydrolysis using beta-glucanase was better than cellulase and glucose recovery of 69 % was realised when beta-glucanase dosage was increased to 10 % (v/w) at 48 h which rose to 73 % at 120 h, releasing 11.19 g/l and 12.17 g/l of glucose respectively. Less than 20 % of glucose was hydrolysed at 10 % (v/w) cellulase at 120 h releasing 2.6 g/l glucose. The optimum experimental condition for hydrolysis of cassava peel was established at 120 h when glucose recovery increased to 88 % for enzyme mixture of 5 % (v/w) cellulase + 10 % (v/w) beta-glucanase producing 14.67 g/l glucose in the hydrolysate.

## Introduction

The call for the use of more efficient material for bioconversion into value added product has shifted the focus in recent years towards more efficient utilization of agroindustrial by-products. In addition, the bioprocessing of these residues can help reduce environmental problems associated with their disposal (Pandey et al. 2000). The exploration of novel, efficient bioprocesses for underused biomasses is thus at the forefront of biotechnological research and industrial application (Rattanachomsri et al. 2009). Cassava is a root crop that produces high yield with minimum input. Yield as high as 45 tons/ha has been reported (Zhang et al. 2003).

Cassava (*Manihot esculenta Cranz*) is the world’s sixth most important crop and is grown in many African, Asian, and Latin American countries. Cassava is a root tuber containing starch and is one of the most important sources of calories in the tropics. Cassava is also widely used as a raw material in many industries to produce animal feed and starch, and more recently for production of ethanol. Cassava can be cultivated on arid and semiarid land where other crops, such as corn, do not grow well (Lin et al. 2011; Pandey et al. 2000).

As a method of wading off attack by predators, cassava produces two cyanogenic glucosides; linamarin and a small amount of lotaustralin (methyl linamarin). These cyanogens are mostly distributed throughout the plant, with large amounts in the leaves and the root cortex (skin layer), and generally smaller amounts in the root parenchyma (interior) (Cardoso et al. 2005).

Fresh cassava does not last long in storage because of its high moisture content. Therefore cassava is usually processed traditionally to obtain different relatively shelf stable intermediate and final products. A traditional method is used and in all cassava is hand peeled. Small scale industrial processing machines are also used in cassava processing. In 2011, 14,240,000 MT of cassava was produced and processed, out of which generated 3,802,080 MT of cassava peels (Bayitse et al. 2013). These peels could make up to about 10 % of net weight of the roots and contain toxic cyanogenic glycosides (Ofoefule and Uzodinma 2009). Peels normally consist of the thin pericarp and the thicker ring. Most processes remove both the pericarp and the thicker ring along with some pulp adhered to the peels. Analysis of the chemical composition of cassava peels indicates the following chemical composition: dry matter 86.5–94.5 %; organic matter 81.9–93.9 %; crude protein 4.1–6.5 %; hemicellulose and cellulose 34.4 %; and lignin 8.4 % (Kongkiattikajorn and Sornvoraweat 2011).

Cellulose is a linear condensation polymer consisting of D-anhydroglucopyranose joined together by β-1,4-glycosidic bonds (Zhang and Lynd 2004). Enzymatic hydrolysis of cellulose is generally described as a heterogeneous reaction system in which cellulases in an aqueous environment react with the insoluble, macroscopic and structured cellulose, containing highly ordered and less ordered regions (Arantes and Saddler 2010). The rate of cellulose hydrolysis mediated by fungal cellulases is typically 3–30 times faster for amorphous cellulose as compared to high crystalline cellulose (Lynd et al. 2002).

One of the typical functions of enzyme is to speed up the rate of enzymatic reaction. However, it has been well-established that in enzymatic hydrolysis of lignocellulosic biomass that the final sugar concentration in the hydrolysis reaction is affected by the enzyme loading under constant substrate concentration (Bommarius et al. 2008). This phenomenon is not only observed in hydrolysis of lignocellulosic biomass, where lignin inhibits the reaction, but on pure cellulosic substrates, such as filter paper and avicel as well. The role of catalyst in any reaction system is to change the rate of the reaction, but not the equilibrium. Therefore, thermodynamically, the enzyme loading should affect the reaction rate, but not the final sugar concentration under constant substrate loadings. Different explanations, such as thermal instability of enzymes, product inhibition, change of the substrate structure into a more recalcitrant form, and deactivation of enzymes by shear stress from agitation have been proposed as possible causes (Taneda et al. 2012).

β-glucosidase is an enzyme which catalyses the hydrolysis of compounds with β-d-glucosidic linkages (Shewale 1982). It has been established that cellulase enzyme complex is made up of three different enzymes (exo β-1,4-glucanase, endo-β-1,4-glucanase and β-glucosidase) which work together to hydrolyse crystalline cellulose. Endoglucanases starts cellulose hydrolysis process, disrupting internal β-1,4-glucosidic bonds along the cellulose chain, increasing the number of ends available for exoglucanases (Shewale 1982). These hydrolysis reactions occur in the amorphous regions of cellulose. Exoglucanases may then cleave off two units of cellobiose from each end of these shorter cellulose chains (Ogeda et al. 2012). Cellobiose and higher cellodextrins are produce when endo-glucanase is used for a long time. The cellobiose and cellodextrins are finally hydrolysed by β-glucosidase to glucose. β-glucosidase plays a major role in cellulose hydrolysis by removing cellobiose which inhibits the action of exo and endo glucanases (Shewale 1982).

Cassava is rich in starch, with competing demand as industrial raw material as well as food. Previous studies on cassava starch focused on starch hydrolysing enzymes such as α-amylase, amyloglucosidase, and pectinase to achieve maximum hydrolysis efficiency of about 98 %, resulting in 160 g/L of total reducing sugar (Collares et al. 2012). Recently, Olanbiwoninu and Odunfa (2015) hydrolysed cassava peel into fermentable sugars using organic acid pre-treatment before enzyme hydrolysis. This process could add additional cost to the fermentation process. However the potential of utilizing enzyme combinations and non-chemical pre-treatment to convert cassava peel to fermentable sugars especially glucose to the knowledge of authors has not been investigated.

The main objective of this research is to investigate and optimise enzymatic hydrolysis of cassava peel using different cocktail of enzymes with no chemical pre-treatment to produce fermentable sugars with emphasis on glucose because of its importance as a base material in biorefinery.

## Materials and methods

### Feedstock preparation

Cassava peel was sampled from a small scale cassava processing plant from Bawjiase, Ghana. The cassava peel was soaked in water for 30 min. This allows easy removal of the brown skin by peeling with the finger. The cassava peel was then dried at 60 °C overnight, and milled.

### Enzymes

Cellulases (NS 22186) and beta-glucanase (NS81223) were kindly provided by Novozymes (A/S, Denmark). NS22186 is a commercial mixture of cellulases, and NS81223 is a commercial endo-β-1, 3(4)-glucanase

### Biomass composition analysis

Dry Matter content (DM) of the sample was measured using the protocol from Enzyme Lab of DTI (Denmark), in principle by weighing the samples before and after overnight drying at 105 °C in oven. Ash content was determined according to the protocol from Enzyme Lab of DTI (Denmark), in principle by weighing before and after ashing at 550 °C for 2 h in Muffle Furnace.

Carbohydrate composition was determined according to the protocol A0003 from Enzyme Lab of DTI (Denmark), in principle by hydrolysing monomer sugars by two steps acid hydrolysis of cassava peel biomass by 72 % (w/w) H_2_SO_4_ at 30 °C for 60 min followed with 4 % (w/w) H_2_SO_4_ hydrolysis at 121 °C for 60 min. The hydrolyzed monosaccharides were then quantified by a HPLC system using refractive index detector equipped with an Aminex HPX-87H column (BioRad Laboratories Ltd., USA) running at 63 °C with 4 mM H_2_SO_4_ as eluent with a flow rate of 0.6 ml/min. Klason lignin content was determined as the ash free residue after the two step hydrolysis.

Protein content was measured in Eurofins Steins Laboratorium A/S (Denmark) in principle by oxidation and conversion of nitrogen in the material to ammonia, by excess sulfuric acid following with excess sodium hydroxide. The amount of ammonia was determined by distilling into an excess boric acid, followed by titration with hydrochloric acid. The content of nitrogen was then calculated, based on the determined ammonia amount. The protein content was afterwards calculated by multiplying the nitrogen content of the sample with a factor of 6.25.

Cyanide content in the cassava peel biomass was measured in Eurofins Steins Laboratorium A/S (Denmark), in principle by appropriate enzymatic hydrolysis, followed by distillation in an acid ambient. The Hydrogen cyanide distillate trapped in a basic ambient was then determined by GC.

Total starch analysis was carried out using Megazyme starch assay kit based on the use of thermostable α-amylase and amyloglucosidase (McCleary et al. 1997; Megazyme International 2014). This method has been adopted by AOAC (Official Method 996.11) and AACC (Method 76.13.01)

### Enzymatic hydrolysis

About 0.2 g of cassava peel samples were weighed into 15 ml falcon tubes. In total 10 ml of 0.2 M acetate buffer (pH 5.0) and milli-Q water at ratio of 2:3 was added. 150 µg/mL Ampicillin (Sigma-Aldrich, Co. LLC.) was added to prevent possible contaminations during the hydrolysis process. 12 groups of different enzyme loadings were run for the investigation as shown below: (1) cellulase (NS22186) (2.5 % v/w-biomass), (2) cellulase (NS22186) (5.0 % v/w-biomass), (3) cellulase (NS22186) (10.0 % v/w-biomass), (4) beta-glucanase (NS81223) (2.5 % v/w-biomass), (5) beta-glucanase (NS81223) (5 % v/w-biomass), (6) beta-glucanase (NS81223) (10 % v/w-biomass), (7) cellulase (NS22186) and beta-glucanase (NS81223) (2.5 % v/w-biomass and 2.5 % v/w-biomass) (8) cellulase (NS22186) and beta-glucanase (NS81223) (5 % v/w-biomass and 2.5 % v/w-biomass), (9) cellulase (NS22186) and beta-glucanase (NS81223) (10 % v/w-biomass and 2.5 % v/w-biomass), (10) cellulase (NS22186) and beta-glucanase (NS81223) (2.5 % v/w-biomass and 10 % v/w-biomass), (11) cellulase (NS22186) and beta-glucanase (NS81223) (5 % v/w-biomass and 10 % v/w-biomass), (12) cellulase (NS22186) and beta-glucanase (NS81223) (10 % v/w-biomass and 10 % v/w-biomass). Incubation of substrates without enzyme addition was set as blank control. Hydrolysis was run at temperature of 50 °C at vertical rocking speed of 30 cycles per minute in Environ Geni incubator (Scientific Industries Inc.) for 120 h. Glucose concentrations were measured by a high performance liquid chromatography (HPLC) system using refractive index detector equipped with an Aminex HPX-87H column (Bio-Rad Laboratories Ltd., USA) running at 63 °C with 4 mM H_2_SO_4_ as eluent with a flow rate of 0.6 ml/min.

### Calculations

The efficiency of enzymatic hydrolysis was evaluated by the recovery of glucose which is the focus of this study. Glucose recovery is defined as the total amount of glucose in the hydrolysate compared with the total glucose amount existed in biomass, following the equation:

$${\text{Glucose}}\,{\text{recovery}} = \frac{{{\text{glucose}}\,\,{\text{concentration}}\,{\text{in}}\,{\text{the}}\,{\text{hydrolysate}}\,({\text{g}}/{\text{l}}) \times \,{\text{volume}}\,{\text{of}}\,{\text{hydrolysate}}\,({\text{l}})}}{{{\text{glucose}}\,\,{\text{concentration}}\,(\% {\text{DM}}) \times {\text{sample}}\,{\text{for}}\,{\text{hydrolysis}}\,({\text{g}}) \times \,{\text{DM}}\,{\text{of}}\,{\text{the}}\,{\text{sample}}}} \times 100\,\%$$

## Results

### Biomass composition

The chemical composition of cassava peel as analysed is given in Table 1. About 83 % DM of the cassava peel was glucose whiles xylose and arabinose have taken only minor amount of 2.31 and 2.35 % respectively. The residual starch component of the cassava peel was 47.16 %. The protein was 2.40 % and 9.3 mg/kg cyanide was recorded.

**Table 1:** Chemical composition of cassava peel

Sample	DM (%)	Ash (%DM)	Lignin (%DM)	Starch (%DM)	Protein (%DM)
Cassava peel	89.70 ± 0.06	6.30 ± 0.34	1.92 ± 0.07	47.16 ± 3.19	2.40 ± 0.28

Sample	Cyanide (mg/kg)	Glucose (%DM)	Xylose (%DM)	Arabinose (%DM)	
Cassava peel	9.30 ± 0.42	83.41 ± 0.82	2.31 ± 0.08	2.35 ± 0.08	

### Enzymatic hydrolysis

**Fig. 1:**
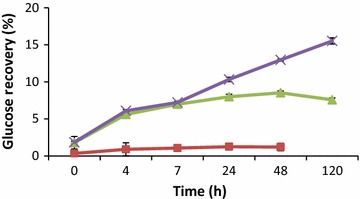
Time course of glucose hydrolysis by addition of three different loadings of cellulase (NS22186) (*square*) 2.5 % v/w cellulase, (*triangle*) 5 % v/w cellulase and (*cross*) 10 % v/w cellulase

**Fig. 2:**
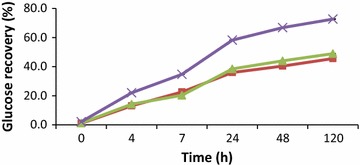
Time course of glucose hydrolysis by addition of three different loadings of beta-glucanase (NS81223) (*square*) 2.5 % v/w beta-glucanase, (*triangle*) 5 % v/w beta-glucanase and (*cross*) 10 % v/w beta-glucanase

Enzymatic hydrolysis of the cassava peel with cellulases generally released least concentration of glucose within the specific time intervals of the experiment, as compared to β-1,3(4)-glucanase and their combinations (Tables 2, 3 and 4). Enzymatic hydrolysis with 2.5 % (v/w) cellulases (NS22186) at 4 h released 0.9 g/l of glucose which rose to 1.2 g/l in 48 h. Increasing the cellulases loading to 5 % did not increase the glucose concentration significantly as compared to 2.5 % (v/w). However, glucose concentration was doubled from 1.02 g/l at 4 h to 2.17 g/l at 48 h of hydrolysis which increased marginally to 2.6 g/l at 120 h when the enzyme loading was increased to 10 % cellulase (Table 2). At the same enzyme loading (v/w), β-glucanase showed a significant higher glucose conversion rate and higher conversion yield than cellulase (Table 2; Figs. 1, 2).

**Table 2:** Glucose concentration (g/l) in the hydrolysate at different cellulase and β-glucanase dosages (2.5, 5, 10 % v/w)

Time (h)	2.5 % (v/w)-biomass		5 % (v/w)-biomass		10 % (v/w)-biomass	
	Cellulase	β-glucanase	Cellulase	β-glucanase	Cellulase	β-glucanase
0	0.34 ± 0.01	0.17 ± 0.02	0.3 ± 0.14	0.21 ± 0.02	0.31 ± 0.02	0.36 ± 0.01
4	0.9 ± 0.15	2.19 ± 0.02	0.94 ± 0.03	2.37 ± 0.03	1.02 ± 0.03	3.69 ± 0.01
7	1.06 ± 0.01	3.79 ± 0.01	1.17 ± 0.03	3.39 ± 0.03	1.21 ± 0.03	5.82 ± 0.02
24	1.25 ± 0.00	6.03 ± 0.03	1.34 ± 0.06	6.42 ± 0.02	1.73 ± 0.05	9.75 ± 0.03
48	1.2 ± 0.08	6.77 ± 0.02	1.43 ± 0.04	7.34 ± 0.04	2.17 ± 0.03	11.19 ± 0.02
120	NA	7.64 ± 0.06	1.27 ± 0.04	8.16 ± 0.02	2.6 ± 0.07	12.17 ± 0.04

**Table 3:** Glucose concentration (g/l) in hydrolysate at different dosages of cellulase (2.5, 5, 10 % v/w) in combination with beta-glucanase dose of (2.5 % v/w) in each mixture

Time (h)	2.5 % (v/w) β-glucanase		
	2.5 % (v/w) cellulase	5 % (v/w) cellulase	10 % (v/w) cellulase
0	0.38 ± 0.02	0.35 ± 0.02	0.65 ± 0.01
4	2.53 ± 0.02	2.21 ± 0.03	2.56 ± 0.01
7	3.45 ± 0.01	3.19 ± 0.03	3.84 ± 0.02
24	7.05 ± 0.03	6.23 ± 0.02	7.17 ± 0.03
48	9.23 ± 0.02	8.41 ± 0.04	9.82 ± 0.02
120	11.31 ± 0.06	10.31 ± 0.02	11.86 ± 0.04

**Table 4:** Glucose concentration (g/l) in hydrolysate at different dosages of cellulase (2.5, 5, 10 % v/w) in combination with beta-glucanase dose of (10 % v/w) in each mixture

Time (h)	10 % (v/w) β-glucanase		
	2.5 % (v/w) cellulase	5 % (v/w) cellulase	10 % (v/w) cellulase
0	0.47 ± 0.02	0.52 ± 0.02	0.95 ± 0.02
4	3.83 ± 0.02	3.97 ± 0.03	4.57 ± 0.02
7	6.32 ± 0.02	6.52 ± 0.01	6.81 ± 0.02
24	10.52 ± 0.03	11.58 ± 0.01	12.76 ± 0.03
48	12.68 ± 0.03	14.36 ± 0.03	12.93 ± 0.01
120	14.07 ± 0.03	14.67 ± 0.01	NA

Mixing cellulase and beta-glucanase at 2.5 % (v/w) each increased glucose concentration from 2.53 g/l at 4 h to 11.31 g/l at 120 h. When cellulase dosage was increased to 5 % in the enzyme mixture at the same β-glucanase dosage there was no increase in glucose concentration. However further increasing cellulase to 10 % (v/w) only produced a marginal increase in glucose concentration from 2.56 g/l at 4 h to 11.86 g/l (Table 3). When the β-glucanase dosage in the enzyme mixture was increased to 10 % (v/w) with 5 % (v/w) cellulase, glucose released reached its maximum in the hydrolysate and glucose concentration ranging from 3.9 g/l at 4 h to 14.67 g/l at 120 h (Table 4).

The efficiency of the enzymatic hydrolysis was evaluated using the calculated glucose recovery of the enzyme loadings with time. Less than 10 % glucose was recovered when 2.5 % (v/w) and 5 % (v/w) cellulase were used respectively. However there was an increase in glucose recovery to 15 % when the dosage was increased to 10 % (v/w) at 120 h of hydrolysis (Fig. 1). There was improvement in glucose recovery when β-glucanase was used as compared to cellulase. 2.5 % (v/w) β-glucanase recorded glucose recovery of 46 % at 120 h whiles 5 % (v/w) β-glucanase yielded 49 % at the same time of hydrolysis. There was a significant improvement in glucose recovery to 69 % when β-glucanase dosage was increased to 10 % (v/w) at 48 h and shot to 73 % at 120 h (Fig. 2).

**Fig. 3:**
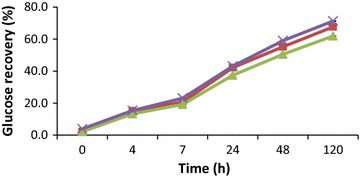
Time course of glucose hydrolysis by addition of three different loadings of cellulase (NS22186) and beta-glucanase (NS81223) mixture. (*square*) 2.5 % v/w cellulase + 2.5 % v/w beta-glucanase, (*triangle*) 5 % v/w cellulase + 2.5 % v/w beta-glucanase and (*cross*) 10 % v/w cellulase + 2.5 % v/w beta-glucanase

**Fig. 4:**
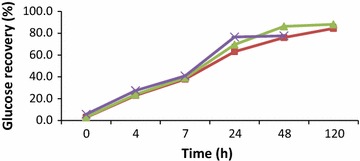
Time course of glucose hydrolysis by addition of three different loadings of cellulase (NS22186) and beta-glucanase (NS81223) mixture. (*square*) 2.5 % v/w cellulase + 10 % v/w beta-glucanase, (*triangle*) 5 % v/w cellulase + 10 % v/w beta-glucanase and (*cross*) 10 % v/w cellulase + 10 % v/w beta-glucanase

When the enzyme mixture had the same dosage of β-glucanase (2.5 % v/w) and cellulase (2.5 % v/w) there was an increase in glucose recovery from 15 % at 4 h to over 3 times at 48 h and subsequently increased over 4 folds at 120 h (Fig. 3). Increasing cellulase dosage in the mixture to 5 % (v/w) did not have any effect on glucose recovery increase. However when the cellulase dosage was further increased to 10 % (v/w) in the mixture there was a 5 fold increase in glucose recovery from 15 % at 4 h to 59 % at 48 h which went up to 71 % at 120 h (Fig. 3). At constant dosage of 2.5 % (v/w) β-glucanase, increasing cellulase dosage to 10 % (v/w) in the mixture did not significantly increase glucose recovery (Fig. 3). By increasing the dosage of β-glucanase to 10 % (v/w) in the enzyme mixture, glucose recovery increased progressively with increasing cellulase dosage recording over 80 % recovery at 120 h of hydrolysis (Fig. 4).

## Discussions

The high glucose content of cassava peel from the composition analysis could partly be attributed to the peeling process which normally leaves some amount of the flesh which contains starch as well as the cellulose content (Table 1). The total cyanide content of cassava parenchyma is dependent on the cultivar, the environment and various other factors. Cyanide levels of between 1 and 1500 ppm can be recorded (Bokanga 1994). The low cyanide content could be due to the variety of cassava (Afisiafi) as well as the pre-treatment process involving soaking in water to remove the brownish skin, and drying at 60 °C for 24 h before milling. Attahdaniel et al. (2013) reduced cyanide level in cassava peel from 268 to 140 mg by drying. Tewe and Iyayi (1989) also reduced cyanide in cassava peel from 364.2–814.7 ppm to 264.3–321.5 ppm by sundrying. They also confirmed that the peel of “bitter” cassava variety was shown to contain 650 ppm whiles the “sweet” variety contained an average of 200 ppm. Cyanide levels up to 1600 mg/kg have been reported in untreated cassava peel (Tivana 2012) The reduction of cyanide content by the above treatment process was significant. Since cyanide is a toxic compound that would affect/inhibit the microbe e.g. yeast fermentation behaviours, this simple pretreatment way claims to be an efficient method for detoxifying the biomass for being processed for further biological conversion, e.g. bioethanol/biobutanol/lactic acid production through fermentation. The low cyanide content reduces the risk of fermentation inhibition which is normally associated with cassava peel as a result of high level of toxic cyanogenic glycosides (Ofoefule and Uzodinma 2009).

The low concentration of glucose released by cellulases during the enzymatic hydrolysis could be due to the composition of the cassava peel which is made up of residual starch, cellulose, hemicellulose and lignin on which the cellulase is only able to convert cellulose to beta-glucose, or shorter polysaccharides and oligosaccharides by hydrolysing the 1, 4-beta-d-glycosidic linkages resulting in less than 16 % of glucose recovery (Fig. 1). However, at the same enzyme loading, there was higher glucose recovery of 73 % (Fig. 2) with corresponding increase in glucose concentration by β-glucanase (Table 2). This phenomenon could be attributed to break down of glucan, a polysaccharide made of several glucose sub-units to release glucose.

The low release of glucose by cellulase during the enzymatic hydrolysis might have affected the overall glucose recovery in the enzyme mixture even when cellulase dosage was increased from 2.5 % (v/w) to 10 % (v/w) (Fig. 3). This phenomenon could be attributed to the release of cellobiose which inhibits the action of exo and endo glucanases (Shewale 1982). This indicated that the cellulase enzyme activity has reached its maximum at 5 % (v/w) and doubling its loading could not be beneficial. Moreover, increasing β-glucanase contributed to overall increase activity of β-glucosidase which works together with other enzymes to hydrolyse crystalline cellulose. The increase in glucose recovery could be due to increase activity of β-glucosidase in the mixture when β-glucanase dosage was increased thereby enhancing the hydrolysis of cellobiose and cellodextrins to glucose. β-glucosidase plays a major role in cellulose hydrolysis by removing cellobiose which inhibits the action of exo and endo glucanases. Consequently, at maximum dosage of cellulase and beta-glucanase mixture there was no significant increase in glucose concentration after 48 h of hydrolysis (Table 4).

In summary, cassava peel which is considered as waste is actually a rich resource for fermentable sugars since it contained substantial amount of residual starch. Enzymatic hydrolysis of cassava peels was evaluated using cellulase and beta-glucanase enzymes and their mixtures at three different enzyme loadings. The efficiency of the hydrolysis using beta-glucanase was better than cellulase and glucose recovery of 69 % was realised when beta-glucanase dosage was used at 10 % (v/w) at 48 h and rose to 73 % at 120 h which released 11.19 g/l and 12.17 g/l of glucose respectively. Less than 20 % of glucose was hydrolysed at 10 % (v/w) cellulase at 120 h releasing 2.6 g/l glucose. The highest glucose recovery was obtained at 88 % through hydrolysis of 13.33 g/l cassava peel for 120 h by enzyme mixture of 5 % (v/w) cellulase + 10 % (v/w) beta-glucanase, producing 14.67 g/l glucose in the hydrolysate. Although glucose recovery was more than 80 %, the use of α-amylase and amyloglucosidase in the enzyme mixture can be explored in future work because of the residual starch.
